# Supplementary material for: Structure of Staphylococcal Enterotoxin N: Implications for Binding Properties to Its Cellular Proteins
Source: Int J Mol Sci. 2019 Nov 25;20(23):5921. doi: 10.3390/ijms20235921 (PMC6928602; doi:10.3390/ijms20235921)
Supplement: Supplementary file 1 [file ijms-20-05921-s001.pdf]

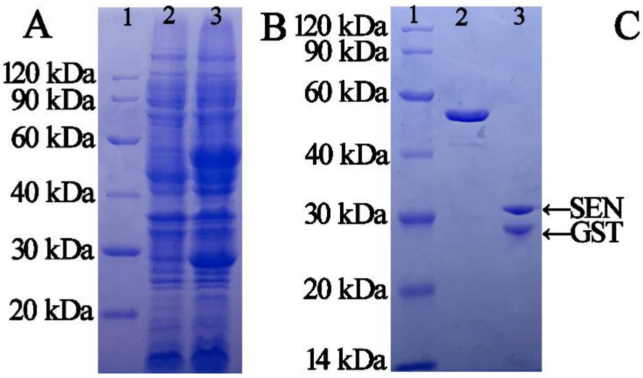

Figure S1. Recombinant SEN production and purification. (**A**) Production of SEN in *Escherichia coli* BL21 (DE3) cells by isopropyl β-D-1-thiogalactopyranoside induction (IPTG). Lane 1: protein molecular weight standards; lane 2: whole cell lysate before IPTG induction; lane 3: whole cell lysate after induced with IPTG. (**B**) GST-fused SEN after GST-affinity chromatography (lane 2) and cleavage of GST tag with TEV protease (lane 3). (**C**) SEN after MonoQ anion exchange chromatography (lane2). (**D**) SEN after purification by Superdex 200 column (lane2).

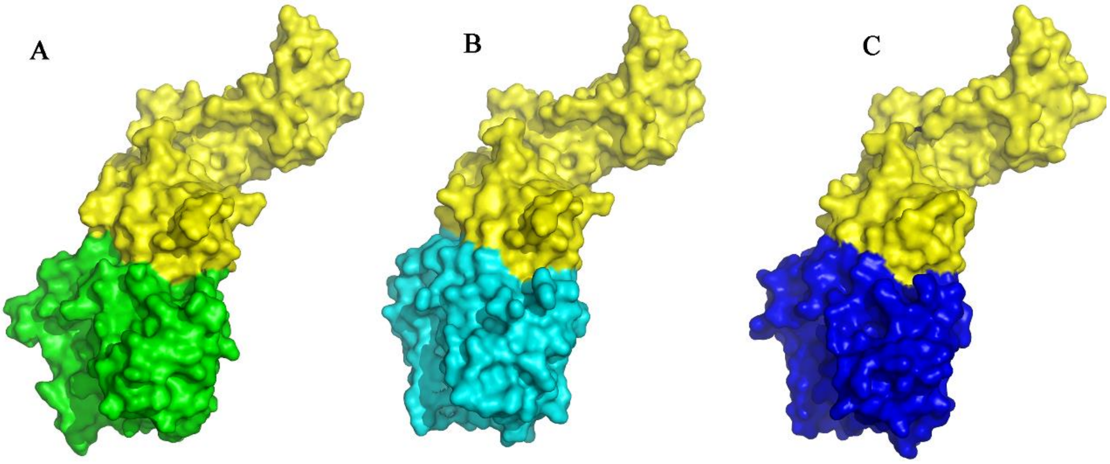

Figure S2. Structural model of SAg in complex with hTRBV7-9. (A) SEN-hTRBV7-9 complex. The complex was obtained by energy minimization. (B) SEA-hTRBV7-9 (PDB entry: 5FK9). (C) SEE-hTRBV7-9 (PDB entry: 5FKA). hTRBV7-9 is shown in yellow. SEN, SEA, and SEE are shown in green, cyan, and blue, respectively.

Table S1. Pairwise RMSD values (Å) between SEN and other SAgs.

Evolutionary group	SAg	PDB entry	Chain ID	Sequence Length	C_α RMSD (Å)
Group I	TSST-1	2QIL	A	194	2.22
	SET	1M4V	A	204	2.23
	SEIX	5U75	A	168	2.51
Group II	SEB	3SEB	A	238	1.39
	SEC	1CK1	A	239	1.68
	SEG	1XXG	A	233	1.65
Group III	SEA	1SXT	A	233	1.13
	SEE	5FKA	C	233	1.23
	SEH	2XNA	C	217	1.38
Group V	SEI	2G9H	D	218	1.67
	SEK	2NTT	A	217	1.55

Table S2. Intermolecular hydrogen bonds in structural model of SEN-hTRBV7-9 and in comparison to that in SEA-hTRBV7-9 (PDB entry: 5FK9) and SEE-hTRBV7-9 (PDB entry: 5FKA) structures.

SEN-hTRBV7-9		SEA-hTRBV7-9		SEE-hTRBV7-9	
SEN	hTRBV7-9	SEA	hTRBV7-9	SEE	hTRBV7-9
Gly113	Glu53	Asn25	Gln55	Asn21	Lys58
Asn114	Glu53	Val174	Gln81	Ser24	Gln55
Lys190	Phe66	Trp63	Ser28	Asn25	Gln55
		Trp63	His30	Gln28	Arg70
		Tyr94	Asn52	Tyr64	Asn52
		Gly93	Glu53	Ser174	Gln81
		Tyr94	Glu53	Trp63	Ser28
		Thr21	Gln55	Trp63	His30
		Asn25	Gln55	Tyr64	Glu53
		Thr21	Leu56	Gly93	Glu53
		Tyr205	Leu56	Tyr64	Glu53
		Tyr32	Glu69	Asn25	Gln55
				Asn21	Leu56
				Tyr205	Leu56
				Asn21	Glu57
				Ser174	Asp64
				Arg27	Ser67
				Arg27	Ala68
				Tyr32	Glu69
				Gln28	Arg70
